# Comparisons of Primary HIV-1 Drug Resistance between Recent and Chronic HIV-1 Infection within a Sub-Regional Cohort of Asian Patients

**DOI:** 10.1371/journal.pone.0062057

**Published:** 2013-06-27

**Authors:** Sasisopin Kiertiburanakul, Romanee Chaiwarith, Sunee Sirivichayakul, Rossana Ditangco, Awachana Jiamsakul, Patrick C. K. Li, Pacharee Kantipong, Christopher Lee, Winai Ratanasuwan, Adeeba Kamarulzaman, Annette H. Sohn, Somnuek Sungkanuparph, for the TREAT Asia Studies to Evaluate Resistance Surveillance and Monitoring Studies

**Affiliations:** 1 Faculty of Medicine Ramathibodi Hospital, Mahidol University, Bangkok, Thailand; 2 Research Institute for Health Sciences, Chiang Mai University, Chiang Mai, Thailand; 3 Faculty of Medicine, Chulalongkorn University and HIV-NAT/Thai Red Cross AIDS Research Centre, Bangkok, Thailand; 4 Research Institute for Tropical Medicine, Manila, Philippines; 5 The Kirby Institute, University of New South Wales, Sydney, Australia; 6 Queen Elizabeth Hospital, Hong Kong, China; 7 Chiang Rai Regional Hospital, Chiang Rai, Thailand; 8 Hospital Sungai Buloh, Kuala Lumpur, Malaysia; 9 Faculty of Medicine, Siriraj Hospital, Mahidol University, Bangkok, Thailand; 10 University Malaya Medical Center, Kuala Lumpur, Malaysia; 11 TREAT Asia, amfAR - The Foundation for AIDS Research, Bangkok, Thailand; Chinese Academy of Sciences, Wuhan Institute of Virology, China

## Abstract

**Background:**

The emergence and transmission of HIV-1 drug resistance (HIVDR) has raised concerns after rapid global antiretroviral therapy (ART) scale-up. There are limited data on the epidemiology of primary HIVDR in resource-limited settings in Asia. We aimed to determine the prevalence and compare the distribution of HIVDR in a cohort of ART-naïve Asian patients with recent and chronic HIV-1 infection.

**Methods:**

Multicenter prospective study was conducted in ART-naïve patients between 2007 and 2010. Resistance-associated mutations (RAMs) were assessed using the World Health Organization 2009 list for surveillance of primary HIVDR.

**Results:**

A total of 458 patients with recent and 1,340 patients with chronic HIV-1 infection were included in the analysis. The overall prevalence of primary HIVDR was 4.6%. Recently infected patients had a higher prevalence of primary HIVDR (6.1% vs. 4.0%, p = 0.065) and frequencies of RAMs to protease inhibitors (PIs; 3.9% vs. 1.0%, p<0.001). Among those with recent infection, the most common RAMs to nucleoside reverse transcriptase inhibitors (NRTIs) were M184I/V and T215D/E/F/I/S/Y (1.1%), to non-NRTIs was Y181C (1.3%), and to PIs was M46I (1.5%). Of patients with chronic infection, T215D/E/F/I/S/Y (0.8%; NRTI), Y181C (0.5%; non-NRTI), and M46I (0.4%; PI) were the most common RAMs. K70R (p = 0.016) and M46I (p = 0.026) were found more frequently among recently infected patients. In multivariate logistic regression analysis in patients with chronic infection, heterosexual contact as a risk factor for HIV-1 infection was less likely to be associated with primary HIVDR compared to other risk categories (odds ratio 0.34, 95% confidence interval 0.20–0.59, p<0.001).

**Conclusions:**

The prevalence of primary HIVDR was higher among patients with recent than chronic HIV-1 infection in our cohort, but of borderline statistical significance. Chronically infected patients with non-heterosexual risks for HIV were more likely to have primary HIVDR.

## Introduction

Highly active antiretroviral therapy (ART) has significantly improved the prognosis of HIV-1-infected patients and prolonged survival worldwide [1]–[3]. Since 2004, the number of people receiving therapy has increased substantially, and exceeded 5 million people in low- and middle-income countries in 2010 [3]. In 2009, UNAIDS reported a 30% increase in the number of people receiving treatment in a single year [3].

The epidemic within the Asia-Pacific region includes largely concentrated epidemics that vary by transmission risk factors. For example, heterosexual transmission is the dominant risk factor for transmission in Thailand, male-to-male sex is the primary risk factor in the Philippines, and injection drug use is the main driver of the epidemic in Malaysia and Indonesia, but Hong Kong has multiple primary epidemic drivers. The main HIV subtype within Southeast Asia is circulating recombinant factor 01, type AE (CRF01_AE). ART was available in high-income countries in the region at similar times to Western countries, including use of protease inhibitors (PI). National programs in resource-limited settings were not scaled up until the mid-2000s, and continue to primarily use non-nucleoside reverse transcriptase inhibitors (NNRTI).

However, now that low- and middle-income countries in the region are increasing their ART coverage [4], [5], there has been an emerging challenge of HIV-1 drug resistance (HIVDR) and first-line treatment failure. Primary HIVDR, pre-existing resistance in those who have not received ART [6], [7], is increasing in settings where ART has been widely available for longer periods of time due to a greater likelihood of acquired resistance-associated mutations (RAMs) in the pool of transmissible virus [5], [8], [9]. The transmission of drug-resistant virus is a growing concern, and has been associated with increased mortality, morbidity, and medical expenditures because of compromising the effectiveness of first-line ART regimens [4], [10], [11].

The reported prevalence of primary HIVDR varies from approximately 1.1% to 21% in the United States, Europe, and Africa [5], [11]–[14]. There are limited data on the epidemiology of primary HIVDR in resource-limited settings in Asia, and pre-ART resistance testing is not routinely performed owing to high cost and limited laboratory infrastructure. To assess the extent of HIVDR in Asia, surveillance of primary HIVDR and monitoring of the development HIVDR in patients taking ART have been conducted through the TREAT Asia Studies to Evaluate Resistance-Surveillance (TASER-S) and the TREAT Asia Studies to Evaluate Resistance-Monitoring (TASER-M) programs [15]. The primary objective of TASER-S is to assess the prevalence of primary HIVDR in ART-naïve, recently HIV-1-infected patients. The primary objectives of TASER-M are to evaluate the prevalence and incidence of emerging HIVDR in ART-naïve HIV-1-infected patients initiating first-line ART and those who are switching from first-line ART to second-line ART after treatment failure.

We aimed to compare the prevalence of primary HIVDR and the distribution of frequencies of RAMs in these cohorts; and to determine factors associated with primary HIVDR. Knowing this epidemiological data can inform healthcare providers and national policy makers in Asia on the emerging issue of primary HIVDR.

## Patients and Methods

Four clinical research sites participated in the surveillance study TASER-S [Thailand (N = 2), Hong Kong (N = 1), and Philippines (N = 1)], and 11 sites participated in the monitoring study TASER-M [Thailand (N = 5), Malaysia (N = 3), Hong Kong (N = 1), Philippines (N = 1), and Indonesia (N = 1)]. ART-naïve HIV-1-infected patients enrolled in these cohorts from 2007 to 2010 were included in this study. All patients provided written informed consent to participate and have their data stored in both the site-level and centralized study databases for the purposes of research.

Recent HIV-1 infection was defined according to the World Health Organization (WHO) 2008 criteria for HIVDR threshold surveys [16]. Briefly, the criteria include 1) laboratory confirmation of HIV-1 infection, 2) evidence of recent infection (i.e., positive BED assay or previous negative HIV-1 test in the past year), or 3) has an indeterminate or negative HIV-1 test with detectable HIV-1 RNA or positive p24 antigen. Only the Philippines site use BED assay for the diagnosis of recent HIV infection. Chronic HIV-1 infection was defined as meeting local or national criteria for ART initiation in treatment-naïve patients. Data were collected on age, sex, ethnicity, HIV-1 exposure, the United States Center for Disease Control and Prevention (CDC) disease stage classification, hepatitis B virus (HBV) and hepatitis C virus (HCV) co-infection status, CD4 cell count, HIV-1 RNA level, and HIV-1 subtype. However, the CDC category and hepatitis co-infection status were collected from the patients in TASER-M cohort only.

Genotypic HIV-1 drug resistance testing was performed locally with externally quality controlled in-house or commercial assays, on samples collected within six months prior to ART initiation. Laboratories were required to participate in the TREAT Asia Quality Assurance Scheme (TAQAS), an external assessment program to build genotyping capacity conducted through the National Serologic Reference Laboratory in Australia [17]. RAMs to the major three drug classes of nucleoside reverse transcriptase inhibitors (NRTIs), non-nucleoside reverse transcriptase inhibitors (NNRTIs), and protease inhibitors (PIs) were assessed using the WHO 2009 list of mutations for surveillance of primary HIVDR [18]. Subtype was determined based on reverse transcriptase and protease sequences submitted for drug resistance interpretation using the Stanford University HIV Drug Resistance Database (version 6.0.11). All sequences were submitted to GenBank and the accession numbers are the following; KC791222–KC791422, KC810320–KC810570, KC810571–KC810821, KC867560–KC867647, KC856944–KC857104, KC857105–KC857265, KC921394–KC921484, KC921485–KC921766, KC921767–KC921992, KC961260, KC962512–KC962549, KC970855–KC970880, KC970881–KC970981, KC970982–KC971006, KC971007, KC971008, KC971009–KC971042, KC994162–KC994341, KC994342–KC994450, KC994451, KC994452, KF059614–KF059717, KF059718–KF059833.

### Statistical analysis

Baseline characteristics, prevalence of primary HIVDR, and RAM frequencies were compared between groups using the Wilcoxon Rank Sum test for continuous variables and Chi-square test or Fisher's exact test for categorical variables. Factors associated with primary HIVDR were assessed using logistic regression analysis. Variables that were selected by univariate analyses at p-value <0.1, as well as those considered *a priori* as possible associated factors on the basis of prior research were included in the final multivariate model using a forward stepwise selection process. Statistical analyses were performed using Stata statistical software version 10.0 (Stata Corporation, College Station, TX, 2007).

## Results

A total of 458 patients with recent and 1,340 patients with chronic HIV-1 infection were included in the analysis. Their baseline characteristics are shown in Table 1. Patients with recent HIV-1 infection were younger (median 23 vs. 36 years old, p<0.001), mostly male (91.9% vs. 65.9%, p<0.001), and less likely to report heterosexual HIV-1 exposure (13.1% vs. 72.2%, p<0.001). They had a lower proportion of infection with CRF01_AE virus (68.8% vs. 78.7%, p<0.001) and higher CD4 cell counts (median 349 vs. 104 cells/mm^3^, p<0.001).

**Table 1:** Baseline characteristics of 1,798 HIV-infected antiretroviral therapy-naïve patients stratified by duration of HIV infection.

Characteristics	Duration of HIV infection		p-value*
	Recent (N = 458)	Chronic (N = 1,340)	
**Age groups, years (%)**			<0.001
≤20	86 (18.8)	12 (0.9)	
21–30	308 (67.2)	295 (22.0)	
31–40	45 (9.8)	552 (41.2)	
41–50	15 (3.3)	333 (24.8)	
≥51	4 (0.9)	148 (11.0)	
**Gender, (%)**			<0.001
Male	421 (91.9)	883 (65.9)	
Female	37 (8.1)	457 (34.1)	
**Ethnicity, (%)**			<0.001
Thai	293 (64.0)	922 (68.8)	
Chinese	67 (14.7)	252 (18.8)	
Filipino	84 (18.3)	40 (3.0)	
Other	14 (3.1)	126 (9.4)	
**HIV exposure, (%)**			<0.001
Heterosexual contact	60 (13.1)	965 (72.2)	
Homosexual contact	369 (80.6)	268 (20.0)	
Injecting drug use	1 (0.2)	51 (3.8)	
Other/unknown	28 (6.1)	56 (4.2)	
**CD4 group, (%)**			<0.001
≤50	13 (2.8)	423 (31.6)	
51–100	6 (1.3)	218 (16.3)	
101–200	24 (5.2)	333 (24.8)	
≥201	263 (57.4)	328 (24.5)	
Missing	152 (33.2)	38 (2.8)	
**HIV RNA group, copies/mL (%)**			<0.001
≤50,000	195 (42.6)	379 (28.3)	
50,001–250,000	120 (26.2)	636 (47.5)	
≥250, 001	60 (13.1)	314 (23.4)	
Missing	83 (18.1)	11 (0.8)	
**Subtype, (%)**			<0.001
CRF01_AE	315 (68.8)	1,055 (78.7)	
B	104 (22.7)	211 (15.7)	
Other, non B	39 (8.5)	74 (5.5)	
**Baseline CDC category, (%)**			
A		641 (47.8)	
B		220 (16.4)	
C		479 (35.8)	
Missing	458 (100.0)	0	
**HBsAg, (%)**			
Negative		1,062 (79.2)	
Positive		131 (9.8)	
Not tested (missing)	458 (100.0)	147 (11.0)	
**Anti-HCV, (%)**			
Negative		968 (72.2)	
Positive		84 (6.3)	
Not tested (missing)	458 (100.0)	288 (21.5)	

**Abbreviations:** HBsAg – hepatitis B surface antigen, anti-HCV – antibody to hepatitis C virus.

* p-value calculated for categorical data that did not include missing values, using Chi-square test or Fisher's exact test.

The crude combined prevalence of primary HIVDR in both cohorts was 4.6% (82 of 1,798 patients). Of these, 28 (6.1%) patients with recent HIV-1 infection and 54 (4.0%) patients with chronic HIV-1 infection had primary HIVDR (p = 0.065). Overall, the frequency of RAMs was 4.1% for NRTIs, 2.3% for NNRTIs, and 1.8% for PIs. Recently infected patients had higher frequencies of RAMs to NRTIs (5.2% vs. 3.6%, p = 0.138), NNRTIs (2.8% vs. 2.2%, p = 0.410), and PIs (3.9% vs. 1.0%, p<0.001).

**Figure 1:**
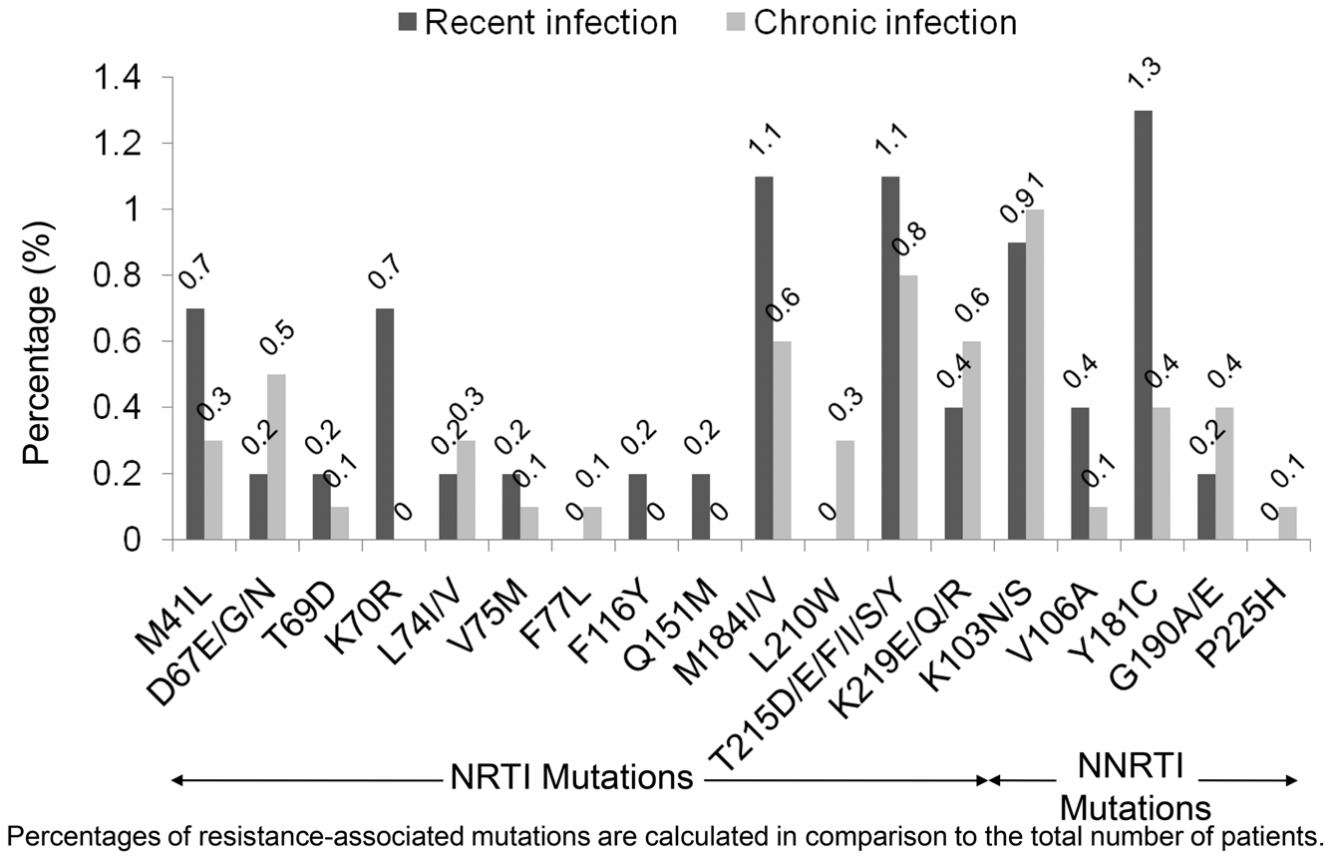
Distribution of reverse transcriptase resistance-associated mutations among 1,798 antiretroviral-naive HIV-infected patients. Abbreviations: NRTI; nucleoside reverse transcriptase inhibitor, NNRTI; non-nucleoside reverse transcriptase inhibitor.

**Figure 2:**
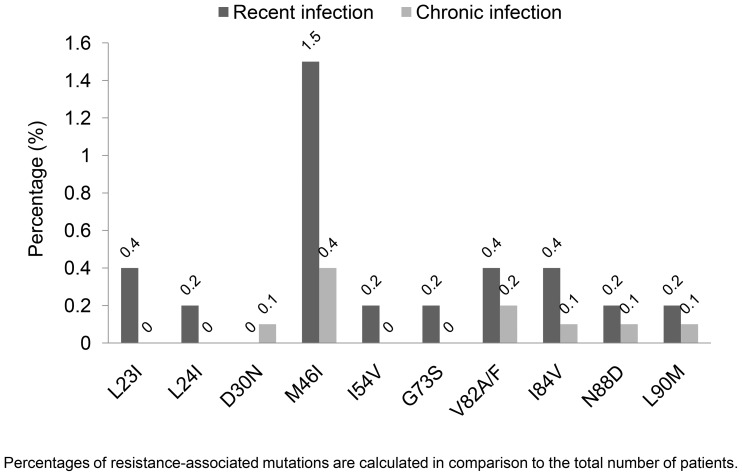
Distribution of protease resistance-associated mutations among 1,798 antiretroviral-naive HIV-infected patients.

The distributions of RAMs among all HIV-1-infected patients are shown in Figures 1 and 2. In patients with recent HIV-1 infection, M184I/V and T215D/E/F/I/S/Y were the most common RAMs to NRTIs (1.1% each); Y181C was the most common RAM to NNRTIs (1.3%), and M46I was the most common RAM to PIs (1.5%). In patients with chronic HIV-1 infection, T215D/E/F/I/S/Y (0.8%), Y181C (0.5%), and M46I (0.4%) were the most common RAMs to the corresponding drug classes. Higher frequencies of K70R (p = 0.016) and M46I (p = 0.026) were present among patients with recent HIV-1 infection; there were no statistically significant differences in the frequencies of other RAMs.

Patients who had primary HIVDR were less likely to report heterosexual sex as their main risk factor for HIV infection (34.2% vs. 58.1%, p<0.001) or be infected with CRF01_AE virus (65.8% vs. 76.7%, p = 0.024). In the unadjusted model of recent infection, no significant factor associated with primary HIVDR was indentified. Among patients with chronic infection, those with heterosexual HIV risk exposure were less likely to have primary HIVDR compared to those within other risk categories [odds ratio (OR) 0.34, 95% confidence interval (CI) 0.20–0.59, p<0.001; Table 2]. In multivariate logistic regression among patients with chronic infection, patients with heterosexual HIV risk exposure also were less likely to have primary HIVDR (OR 0.34, 95% CI 0.20–0.59, p<0.001).

**Table 2:** Factors associated with primary HIV drug resistance by duration of HIV infection - univariate logistic regression.

Variables	Recent HIV infection			Chronic HIV infection		
	Odds ratio	95% Confidence interval	p-value*	Odds ratio	95% Confidence interval	p-value*
**Age groups, years**			0.054			0.112
≤30	1			1		
31–40	3.64	1.44–9.20	0.006	0.63	0.33–1.21	0.168
41–50	1.41	0.18–11.29	0.746	0.55	0.25–1.18	0.125
≥51	6.58	0.65–66.25	0.110	0.44	0.15–1.34	0.151
**Gender**						
Female	1			1		
Male	2.47	0.32–18.69	0.382	1.24	0.68–2.25	0.480
**Ethnicity**						
Non Thai	1			1		
Thai	0.74	0.34–1.60	0.439	0.76	0.43–1.34	0.345
**Exposure**						
Non-heterosexual	1			1		
Heterosexual contact	0.49	0.11–2.13	0.344	0.34	0.20–0.59	<0.001
**CDC category** *						0.589
A				1		
B				1.21	0.57–2.57	0.623
C				0.85	0.46–1.57	0.613
**Subtype**						
Non CRF01_AE	1			1		
CRF01_AE	0.68	0.31–1.50	0.344	0.57	0.32–1.03	0.064
**CD4, cells/mm^3^**			0.394			0.824
≤200	1			1		
≥201	2.54	0.33–19.74	0.373	0.86	0.49–1.66	0.663
Missing	3.06	0.45–28.50	0.225	0.62	0.08–4.59	0.636
**HIV RNA, copies/mL**			0.560			0.508
≤50,000	1			1		
50,001–250,000	0.28	0.06–1.30	0.105	0.75	0.40–1.41	0.377
≥250,001	1.86	0.66–5.26	0.243	0.80	0.38–1.68	0.551
Missing	2.03	0.81–5.11	0.131	2.00	0.24–16.53	0.518
**HBsAg serostatus** *						0.226
Negative				1		
Positive				1.88	0.89–3.98	0.096
Missing				0.90	0.35–2.32	0.827
**Anti-HCV serostatus** *						0.473
Negative				1		
Positive				1.10	0.38–3.15	0.856
Missing				0.63	0.29–1.36	0.238

**Abbreviations:** HBsAg – hepatitis B surface antigen, anti-HCV – antibody to hepatitis C virus.

* p-value is for test for trend (excluding missing values) for age groups, CDC category, and HIV RNA.

## Discussion

To our knowledge, this is the largest cohort study of primary and pre-ART HIVDR among Asian HIV-1-infected patients. The present study showed an overall prevalence of primary HIVDR of almost 5%, which is categorized as low prevalence by WHO criteria. Patients with recent HIV-1 infection had a higher prevalence of primary HIVDR compared to those with chronic HIV-1 infection (6.1% vs. 4.0%), although of borderline statistical significance, and had a higher frequency of RAMs to PIs. This may be explained by both virologic and epidemiologic factors. Resistance testing at the time of HIV-1 transmission is more likely to reveal resistance, as the dominant genetic pattern may revert to wild-type over time and be missed by standard resistance testing in the absence of therapy [19], [20].

Previous reports of primary HIVDR among Asian patients ranged from 0% to 13.8% [8], [9], [21]–[24]. Results have varied by study methodology, route of HIV-1 transmission, duration of HIV-1 infection, HIV-1 subtype, pattern of local ART regimen use, and the reference list of RAMs used to assess the presence of relevant HIVDR mutations [4], [25], [26]. The impact of the reference list is seen when comparing these results to a preliminary report of the same cohort that used International AIDS Society (IAS)-USA 2009 criteria and reported 13.8% overall prevalence of primary HIVDR in a subset of 700 patients with chronic HIV-1 infection [23]. The overall prevalence of HIVDR increased to 10.4% when using the IAS-USA criteria in the present study.

In this study, the prevalence of primary HIVDR in patients with recent infection was higher than that found in previous surveys of primary HIVDR in this region [8], [21], [22], [27]. However, the recent study in Thailand showed that primary resistance among men who have sex with men was approximately 7% [28]. The frequencies of RAMs to NRTIs and NNRTIs found in this study were higher than those to PIs. This pattern of primary HIVDR is comparable to reports from African countries and in other settings where NNRTI-based regimens are more commonly used in the scale-up of ART [5], [29]. There were no statistically significant differences in the frequencies of RAMs to NRTIs and NNRTIs between patients with recent and chronic HIV-1 infection, but RAMs to PIs where significantly more frequent in patients with recent HIV-1 infection, which may reflect the increasing use of PIs as part of second-line regimens.

The mutation selected by lamivudine, M184I/V, and the thymidine analogue mutation of T215D/E/F/I/S/Y were the most common RAMs in patients with recent HIV-1 infection and T215D/E/F/I/S/Y were the most common RAMs in those with chronic HIV-1 infection. T215D/E/I/S confers an increased risk of virologic failure for ART-naïve patients started on regimens including zidovudine or stavudine [30], [31]. The most common RAM to NNRTIs found in this study was Y181C, which is selected by nevirapine or efavirenz [32] and the most widely used “anchor” drugs in low- and middle-income countries. The most common regimen which has been widely prescribed in Asia before 2007 is NNRTI-based regimen, such as stavudine/lamivudine/nevirapine and zidovudine/lamivudine/nevirapine.

We also noted higher frequencies of K70R and M46I in patients with recent HIV-1 infection. Previous studies also have shown that K70R has been one of the most common RAMs observed among ART-naïve patients, and is consistent with the widespread use of zidovudine and stavudine in the region [33], [34]. Its higher frequency reflects the longer periods of antiretroviral drugs use in the study settings [35], [36]. M46I was the most common RAMs to PIs found in this study, in agreement with reports from Europe and South Africa [37], [38]. M46I was identified as a relevant RAM for surveillance of primary HIVDR because it reduces the susceptibility to several PIs even in the absence of other surveillance drug-resistance mutations [39].

Our results raise concerns with regards to the risk of treatment failure among Asian patients. Primary HIVDR is clearly associated with the risk of early virologic failure [10], [11]. Although the prevalence of HIVDR in the chronically infected cohort was below 5%, considered in the low range by the WHO criteria, those with non-heterosexual contact as a risk factor for HIV-1 infection were more likely to have primary HIVDR. This finding may guide the selection of ART-naïve patients who would more likely benefit from resistance testing prior to ART initiation in Asia, where routine primary HIVDR screening is not feasible. Further study to determine the cost-effectiveness of routine primary HIVDR testing prior to ART initiation in specific risk groups in Asia is needed.

Our study may not be directly generalizable across the region. Participating sites were largely in urban referral centers and our findings should be considered in the context of the specific characteristics of the study populations and commonly used ART regimens in the study sites. We have described these data as being in Asian patients, but recognize the intra-regional and inter-ethnic variations across the participating study sites. There were difference of clinical characteristics between patients with recent HIV-1 infection and ones with chronic HIV-1 infection. The patients were enrolled separately from each two cohorts. The difference of primary HIVDR between these two patient groups identified might be associated with the sampling bias. In addition, we appreciate that the BED assay is not considered a reliable indicator of recent infection; however, it is a convenient assay. In general, there were few patients for whom the BED test was used as the primary inclusion criteria. In our study, 84 the Filipino patients (18.3% of patients with recent HIV infection) met inclusion criteria based on their BED results. Unfortunately, we do not have CD4 cell count information and it is possible that some Filipino patients may be misclassified by BED to recent HIV infection if they have CD4 cell count <100 cells/mm^3^. Furthermore, as a limited number of variables were available in the TASER-S cohort, the potential effects of some common demographic characteristics could not be assessed. The small number of patients with primary HIVDR limited the power of some of our statistical analysis, notably with regards to patients with recent HIV-1 infection.

## Conclusions

The overall prevalence of primary HIVDR in Asian HIV-1-infected patients in these cohorts is approximately 5%. The prevalence is somewhat higher among patients with recent HIV-1 infection. Patients with chronic infection with non-heterosexual HIV-1 exposures were at higher risk of having primary HIVDR. Ongoing national and regional HIVDR evaluation is needed to identify higher risk populations who may benefit from targeted pre-ART HIVDR testing in settings with limited resources.
